# Efficacy and safety of low‐dose apatinib in ovarian cancer patients with platinum‐resistance or platinum‐refractoriness: A single‐center retrospective study

**DOI:** 10.1002/cam4.3282

**Published:** 2020-07-06

**Authors:** Wei Chen, Ziting Li, Zhong Zheng, Xiaohua Wu

**Affiliations:** ^1^ Department of Gynecologic Oncology Department of Oncology Fudan University Shanghai Cancer Center Shanghai Medical College Fudan University Shanghai China; ^2^ Department of Obstetrics and Gynecology Minhang Hospital Fudan University The Central Hospital of Minhang District Shanghai China

**Keywords:** angiogenesis inhibitor, apatinib, epithelial ovarian cancer, low dose, platinum‐resistant

## Abstract

**Background:**

This study aimed to evaluate the efficacy and safety of apatinib with a low dose of 250 mg/d in the treatment of platinum‐resistant or platinum‐refractory ovarian cancer patients.

**Methods:**

Patients with platinum‐resistant or platinum‐refractory ovarian carcinoma treated with 250 mg/d apatinib in our institution from November 2016 to December 2017 were retrospectively reviewed. The tumor response and progression were evaluated according to the standard by incorporating the levels of CA125 and Response Evaluation Criteria in Solid Tumors 1.1. CTCAE 4.03 was used to evaluate adverse events (AEs).

**Results:**

Fifty‐two eligible patients were enrolled in per‐protocol (PP) analysis and 65 patients (including 13 lost to follow‐up) were included in the intention‐to‐treat (ITT) analysis. In PP analysis, 18 patients (34.6%) had partial response (PR), 22 patients (42.3%) had stable disease (SD), and the disease control rate (DCR) was 61.5%. Median progression‐free survival (PFS) was 4.0 months (95% CI, 2.83‐5.17 m), and median overall survival (OS) was 25.33 months (95% CI, 17.74‐32.92 m). The objective response rate and DCR for patients in ITT analysis were 27.7% and 49.2%, respectively. The top three treatment‐related AEs were hypertension, hand‐foot syndrome, and leukopenia. Eight patients (15.4%) in PP population had grade 3 treatment‐related AEs. Previous chemotherapy lines, number of recurrences, and AEs did not affect the efficacy of apatinib. Age older than 60 was associated with higher rates of disease control and prolonged PFS (*P* < .05).

**Conclusion:**

Apatinib 250 mg/d is a feasible treatment in platinum‐resistant or platinum‐refractory epithelial ovarian cancer (EOC) patients.

## Graphical abstract

Apatinib 250 mg/day is a feasible treatment in patients with platinum‐resistant or platinum‐refractory EOC.

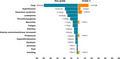

## 1 INTRODUCTION

Epithelial ovarian cancer (EOC) remains the common cause of mortality in female cancer worldwide,^1^ as most patients are diagnosed at an advanced stage. Cytoreduction and platinum‐based chemotherapy are still standard treatments for newly diagnosed ovarian cancer.^2^ Even with improved surgical skills and a high initial response to paclitaxel plus carboplatin chemotherapy, up to 80% of patients with advanced ovarian carcinoma will develop recurrence or are intrinsic resistance to this treatment (defined as platinum‐refractory).^2^, ^3^ Patients, known as platinum‐resistant, who recur within 6 months of the last chemotherapy have poorer progress than those remain disease‐free for more than 6 months (termed platinum‐sensitive).^4^ Besides, patients with platinum‐refractory ovarian cancer or platinum‐resistant diseases have poor responses to alternative single‐agent chemotherapy. The development of more antitumor therapy is particularly necessary. Recently, maintenance treatment with poly ADP‐ribose polymerase inhibitors (PARPi) has gained momentum in platinum‐sensitive recurrent EOC with prolongation of progression‐free survival (PFS).^5^, ^6^ However, its effectiveness in platinum‐resistant patients remains to be investigated. Therefore, antiangiogenesis therapy has become one of the promising treatments for those patients.

Several antiangiogenesis regimens were shown to be effective in recurrent EOC. Bevacizumab, which is a recombinant humanized monoclonal IgG1 antibody that targets vascular endothelial growth factor‐A (VEGF‐A), is approved in the treatment of ovarian cancer.^7^ Apatinib, an oral VEGF receptor‐2 inhibitor, is undergoing phase II/III clinical trials in China and has shown to be effective in a variety of cancer types, such as breast cancer,^8^ non‐small cell lung cancer,^9^ hepatocellular carcinoma,^10^ and ovarian cancer.^11^ A phase II trial in patients with recurrent EOC, which has demonstrated that apatinib has potential antitumor activity in these patients, was published in *Gynecologic Oncology* in 2018.^12^

Various doses of apatinib have been reported in different studies. In a phase III study of stomach/gastroesophageal junction cancer, oral apatinib was administered at 850 mg daily.^13^ However, as a result of toxicity associated with the 750‐mg dose in a phase IIa study in breast cancer, an initial dose of 500 mg/d was recommended.^8^ The previous two studies in ovarian cancer showed an efficacy of 500 mg/d apatinib alone or in combination with chemotherapy,^12^, ^14^ in which dose reductions related to AEs occurred in 82% of the patients for apatinib combined with chemotherapy. In several studies, the use of 250 mg/d is effective.^15^, ^16^ Considering the tolerability of apatinib for those heavily treated ovarian cancer patients, we retrospectively reviewed the efficacy and safety of an initial dose of apatinib at 250 mg/d in platinum‐resistant or platinum‐refractory EOC.

## 2 MATERIALS AND METHODS

### 2.1 Patients and eligibility criteria

This study was approved by the ethics committee of Fudan University Shanghai Cancer Center, and informed consent was exempted by the Ethics Committee because of the retrospective nature of this research. All individual participants consented to the use of their medical records for research purposes. Patients with platinum‐resistant or platinum‐refractory EOC treated with apatinib between November 2016 and December 2017 at our institution were enrolled. The inclusion criteria included the following: (a) The histologic diagnosis was primary EOC, cancer of the fallopian tube, or peritoneal cancer. (b) Patients with at least one measurable lesion according to Response Evaluation Criteria in Solid Tumors (RESIST) criteria (1.1) or patients with a disseminated disease that is not measurable by traditional radiological criteria as peritoneal carcinomatosis or ascites, who have a CA125 level more than twice the laboratory normal value. (c) Patients had platinum‐resistance which is defined as progression within 6 months after the last platinum treatment, and patients had platinum‐refractory which is defined as progression during the initial platinum‐based treatment. (d) Patients were treated with 250 mg of apatinib daily for one cycle (4 weeks) at least. The exclusion criteria are as follows: (a) Concurrent use of other anticancer therapy; (b) Lost to follow‐up or insufficient data for analysis; and (c) concurrent second to primary cancer.

### 2.2 Treatment and dose modification

Initially, patients were administered with a cycle of 250 mg of apatinib once daily for 4 weeks. Patients were temporarily stopped taking apatinib when they experienced grade 3 hematological toxicities or non‐hematologic adverse events (AEs), such as hypertension, hand and foot syndrome, proteinuria, etc They would retreat with dose reduced to 125 mg daily after recovery to grade 1 AE. Apatinib administration was discontinued until unacceptable toxicity after a dose reduction or disease progression. We followed the patients until the time of disease progression, death, discontinuation of treatment, or the cutoff date of 15 March 2019.

### 2.3 Assessment of efficacy and AEs

AEs were graded according to the National Cancer Institute Common Terminology Criteria for Adverse Events (version 4.03). We continuously monitored AEs during monthly follow‐up and throughout the treatment period. The tumor response and progression were evaluated according to the standard by incorporating RECIST 1.1 and the levels of CA125 in serum, as suggested by the Gynecological Cancer Intergroup.^17^ Disease control rate (DCR) was the percentage of patients who achieved the best response of complete response (CR) or partial response (PR) or stable disease (SD) for at least one cycle. PFS was defined as the interval from the start of taking apatinib to disease progression or death. Overall survival (OS) was defined as the duration from the beginning of the treatment to the time of death for any cause.

### 2.4 Statistical analyses

The median (range) and the number of patients (percentage) were used to present quantitative data. Survival analysis was assessed using the Kaplan‐Meier method. Categorical variables were compared using the Chi‐square test and Fisher's exact test. The logistic regression was used for multivariate analysis of independent factors of apatinib response. The cox regression was used for multivariate analysis of PFS and OS. The Statistical Package for the Social Sciences software (SPSS) version 24.0 was used for all statistical analyses. All *P* values reported were two‐tailed, and *P* < .05 was considered statistically significant.

## 3 RESULTS

**FIGURE 1:**
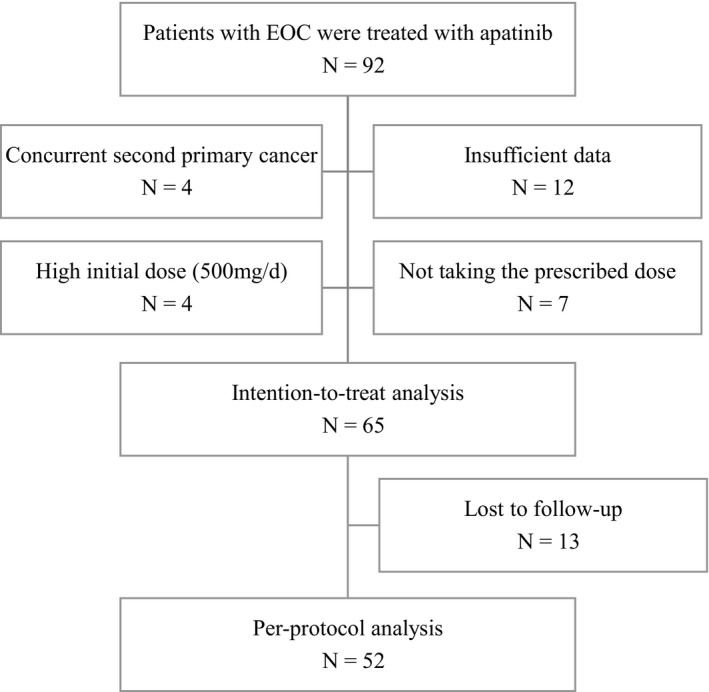
Study algorithm

Ninety‐two patients with EOC were treated with apatinib between November 2016 and December 2017. Four patients were excluded for concurrent second primary cancer. Four patients treated with a high initial dose of apatinib (500 mg/d) and six patients who self‐adjusted the dose were excluded. One patient stopped using apatinib because of a fever. Twelve patients were excluded because their previous treatment was not performed in our hospital and detailed records were lacking. Thus, 65 patients were included in the intention‐to‐treat (ITT) analysis. Thirteen patients were lost to follow‐up. Consequently, a total of 52 EOC patients were included in the per‐protocol (PP) analysis (Figure 1). The main clinicopathological characteristics of patients in PP analysis and patients lost to follow‐up are shown in Table 1. The median age of patients in PP analysis was 55.5 (range, 28‐73) years. The tumor grade of all patients was poorly differentiated. More than half of the patients in PP analysis faced more than two recurrences and 63.4% of them had received ≥ 3 previous lines chemotherapy in addition to primary surgery or secondary cytoreduction before apatinib was administered. Most types of recurrence were diffuse and immeasurable. There were no differences in all the clinicopathological characteristics between patients in PP analysis and patients lost to follow‐up.

**TABLE 1:** Patients' characteristics

Characteristics	N (%)	
	Per‐protocol population	Lost to follow‐up population
Total patients	52	13
Median age (range, y)	55.5 (28‐73)	55.0 (43‐69)
Age (median)		
<60	33 (63.5)	9 (69.2)
≥60	19 (36.5)	4 (30.8)
Histological type		
High‐grade serous carcinoma	48 (92.3)	12 (92.3)
Clear cell carcinoma	2 (3.8)	0
Endometrioid adenocarcinoma	2 (3.8)	1 (7.7)
FIGO stage for first‐line treatment		
IC	3 (5.8)	0
IIB	7 (13.5)	2 (15.4)
IIIA	2 (3.8)	1 (7.7)
IIIB	1 (1.9)	1 (7.7)
IIIC	31 (59.6)	9 (69.2)
IVB	5 (9.6)	0
NA	3 (5.8)	0
Prior lines of chemotherapy		
<3	19 (36.5)	1(7.7)
3‐6	30 (57.7)	10 (76.9)
>6	3 (5.8)	2(15.4)
Regimen of last chemotherapy		
Platinum‐based	33 (63.5)	10 (76.9)
Non‐platinum‐based	19 (36.5)	3 (23.1)
Platinum status		
Refractory	11 (21.2)	1(7.7)
Resistant	41 (78.8)	12 (92.3)
Symptomatic		
No	16 (30.8)	5 (38.5)
Yes	36 (69.2)	8 (61.5)
Measurable lesion		
Yes	11 (21.2)	5 (38.5)
No	41 (78.8)	8 (61.5)

Abbreviations: FIGO, International Federation of Gynecology and Obstetrics; NA, Non‐available.

All patients included in PP analysis were initially administered a dose of 250 mg/d. One patient was reduced to 125 mg daily for 6 months. Interruption of treatment occurred in two patients, and six discontinued apatinib treatments due to intolerable toxicity (grade 3 AE). The median follow‐up time from apatinib treatment to data analysis was 19.2 months (range 1.03‐28.8 m). All patients had discontinued their medication at the time of data analysis. The reasons for discontinuation of apatinib included disease progression (71.4%), grade 3 AEs (15.4%), receiving other therapy (5.6%), lost to follow‐up (1.9%), and death (5.7%).

**FIGURE 2:**
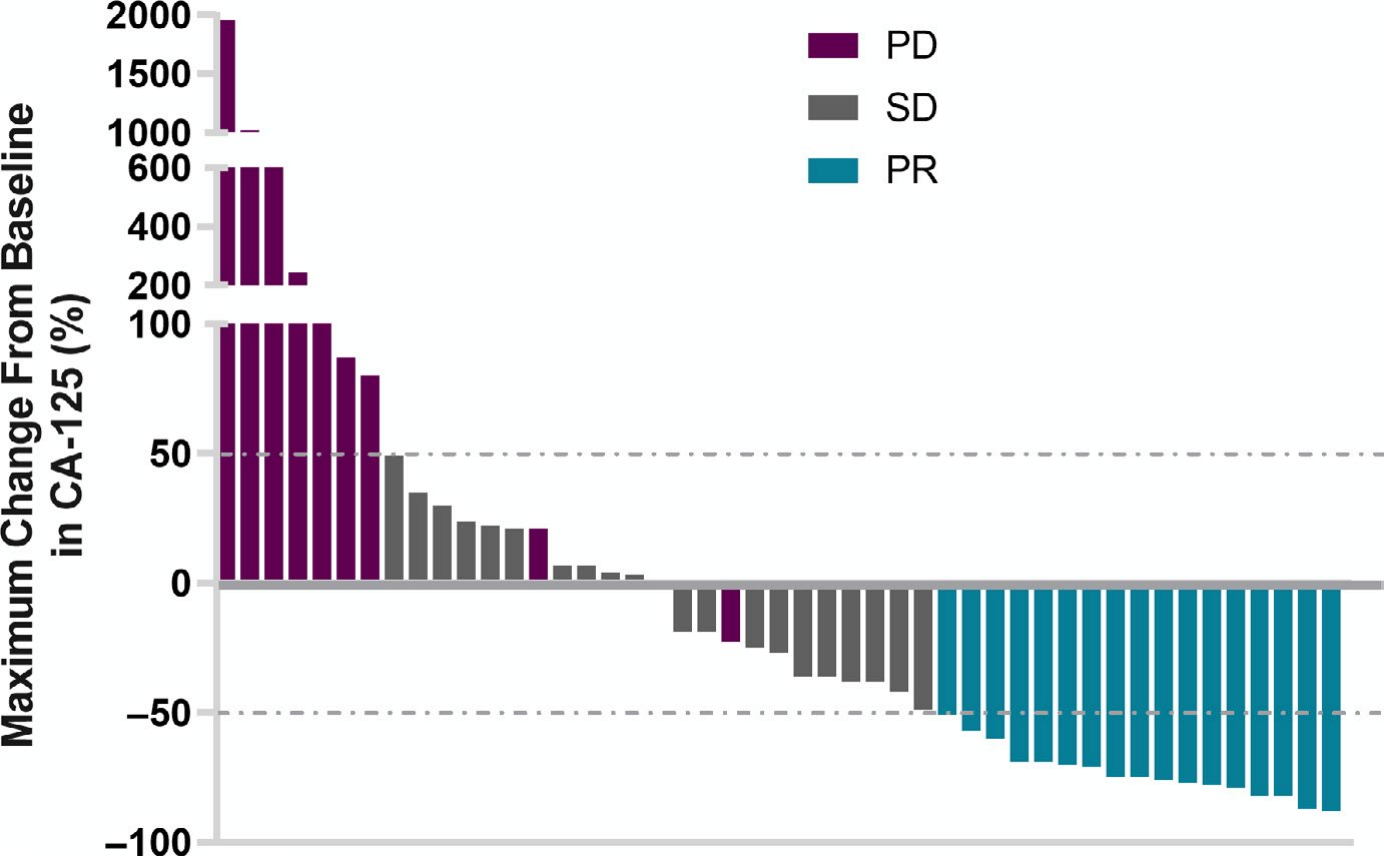
Best percentage changes from baseline in serum CA‐125 levels (N = 47, five patients with normal levels of CA‐125 were not shown). PR partial response, SD stable disease, PD progression disease

**FIGURE 3:**
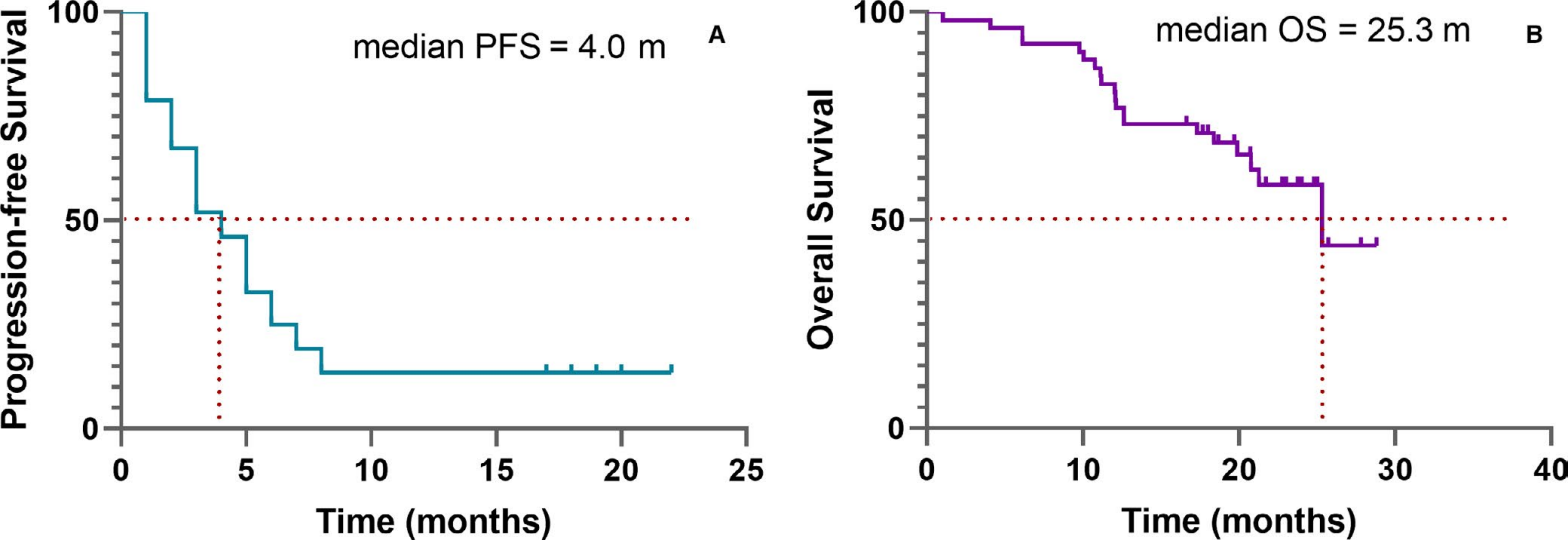
Kaplan‐Meier estimates of progression‐free survival (A) and overall survival (B) of patients who received apatinib treatment (N = 52)

Among 52 patients in PP analysis with an evaluable best response, 18 patients (34.6%) achieved PR and 22 patients (42.3%) had SD. This resulted in a DCR of 61.5% (Table 2). The tumor response in serum CA‐125 levels is shown in Figure 2. Median PFS was 4.0 months (95% confidence interval (CI), 2.83 to 5.17 m) and median OS was 25.33 months (95% CI, 17.74‐32.92 m). The Kaplan‐Meier analysis of PFS and OS is shown in Figure 3. To balance the selection bias, we conducted ITT analysis including those patients who were lost to follow‐up. The DCR for patients in ITT analysis was 49.2%, and the objective response rate (ORR) was 27.7% (Table 2).

**TABLE 2:** Tumor response

Response	No.	Intention‐to‐treat population (N = 65)		Per‐protocol population (N = 52)	
		%	95%CI	%	95%CI
CR	0	0		0	
PR	18	27.7	16.5‐38.9	34.6	21.2‐48.0
SD	22	33.8	22.0‐45.7	42.3	28.4‐56.2
PD	12	18.5	8.8‐28.2	23.1	11.2‐34.9
ORR	18	27.7	16.5‐38.9	34.6	21.2‐48.0
DCR^a^, ^*^	32	49.2	36.7‐61.7	61.5	47.9‐75.2

Abbreviations: CR, Complete response; DCR, Disease‐control rate; ORR, Objective response rate; PD, Progression disease; PR, Partial response; SD, Stable disease.

^a^ The disease control rate was the percentage of patients who had a best response rating of complete or partial response or stable disease that was maintained for at least 1 mo after the first demonstration of that rating on independent review.

**FIGURE 4:**
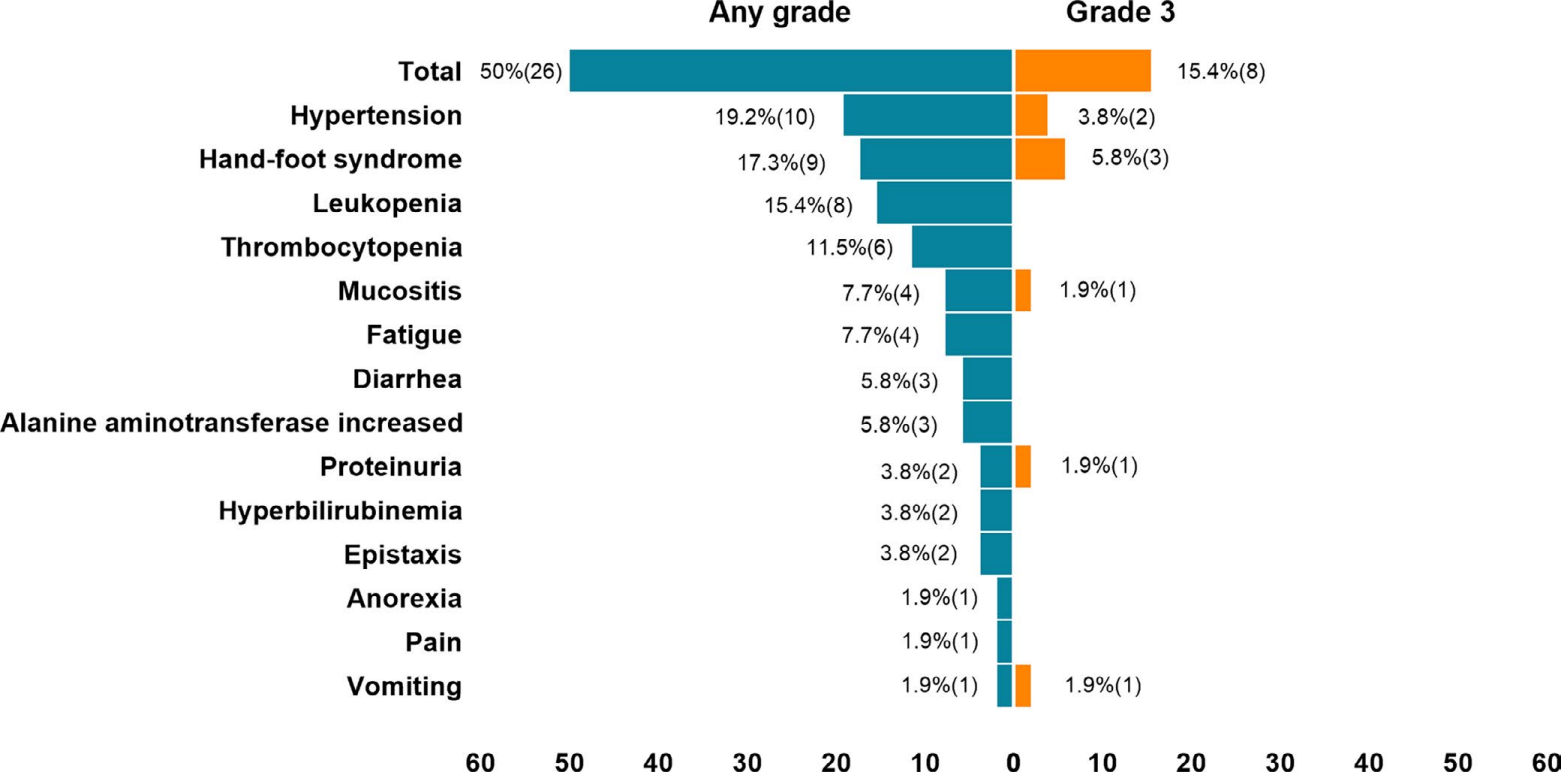
Percentage of patients with any grade or grade 3 adverse events

AEs that occurred in treatment are shown in Figure 4. There was no treatment‐related death. In the PP analysis population, 15.4% (8/52) of the patients experienced grade 3 treatment‐related AEs. The AEs were hand‐foot syndrome (n = 3), hypertension (n = 2), and proteinuria, mucositis oral, and vomiting (n = 1 for each AE). Two patients (3.8%) experienced dose interruption for 2 weeks because of hypertension (n = 1) and epistaxis (n = 1). Overall, adverse reactions accounted for 50.0% (26/52).

**FIGURE 5:**
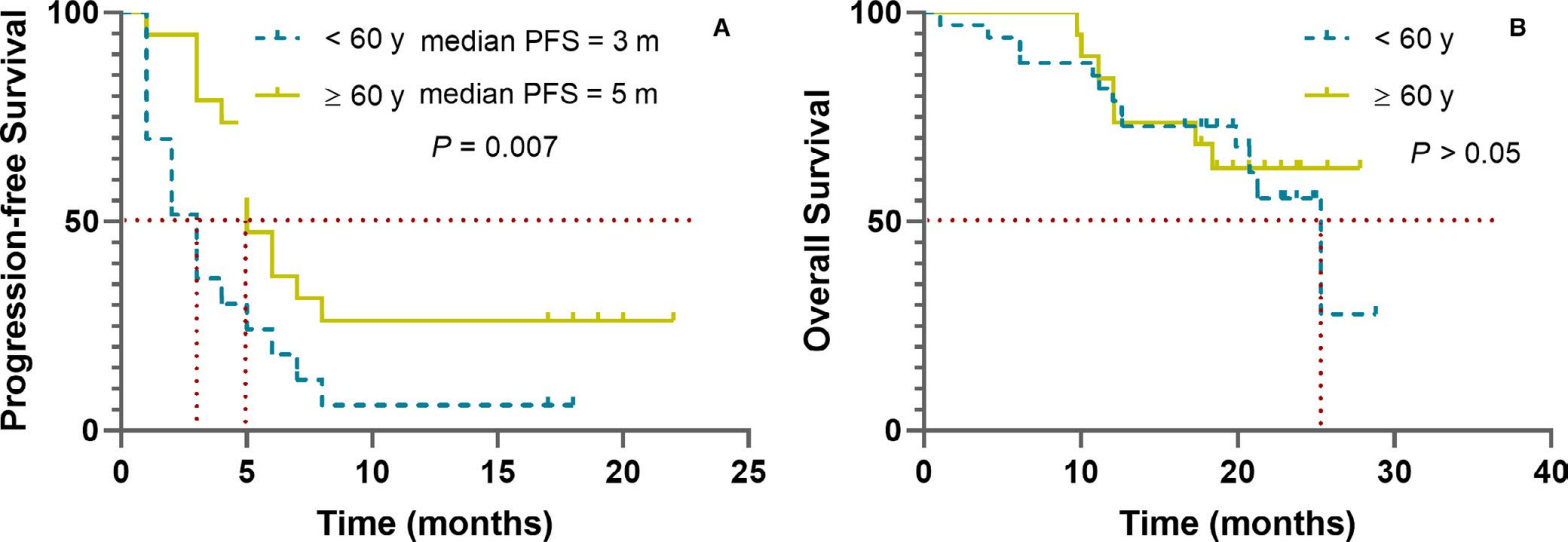
Kaplan‐Meier estimates of progression‐free survival (A) and overall survival (B) of patients who received apatinib treatment stratified by age more than 60 y (yellow) or less (blue)

Next, we analyzed which factors associated with the response of apatinib. As shown in Table 3, prior chemotherapy lines, number of recurrences, and AEs were not associated with the DCR of apatinib. Both univariate and multivariate analyses showed that the DCR of apatinib was only associated with age. It was more effective in patients older than 60 years of age (*P* = .011). The Kaplan‐Meier analysis of PFS and OS by age group is shown in Figure 5. The median PFS of patients over 60 years old (5.0 months, 95%CI: 3.78‐6.22) was longer than that of patients under 60 years old. According to COX multivariate analysis, age was an independent favorable factor of longer PFS (HR = 0.457 (95%CI: 0.241‐0.865)). But none of the above factors were associated with OS in both univariate and multivariate analyses.

**TABLE 3:** DCR in different factors

Type	DCR (%)	Univariate analysis		Multivariate analysis	
		*X* ^2^	*P* value	*P* value	OR (95% CI)
Age		6.502	.011^a^, *	.011^a^, *	
<60	16/33 (48.5)				
≥60	16/19 (84.2)				8.653 (1.625 to 45.081)
Measurable lesion		.785	.376	.521	
Yes	5/11 (45.5)				
No	27/41 (65.9)				
Symptomatic		.009	.924	0.776	
No	10/16 (62.5)				
Yes	22/36 (61.1)				
Platinum status		.035	.851	0.115	
Refractory	6/11 (54.5)				
Resistant	26/41 (63.4)				
Prior chemotherapy lines		4.418	.107	0.070	
1‐2	9/19 (47.4)				
3‐6	22/30 (73.3)			0.292	
>6	1/3 (33.3)			0.064	
AE					
Hand‐foot syndrome		.612	.434		
No	28/43 (65.1)				
Yes	4/9 (44.4)				
Hypertension		<.001	1.000		
No	26/43 (60.5)				
Yes	6/9 (66.7)				

Abbreviations: AE, Adverse events; CI, Confidence interval; DCR, Disease control rate; OR, Odds ratio.

* *P* values with statistical significance were denoted.

## 4 DISCUSSION

As far as we know, this is the first study to evaluate the efficacy and safety of 250 mg/daily apatinib in patients with platinum‐resistant or platinum‐refractory EOC. Two prospective phase II studies showed promising efficacy of apatinib 500 mg once daily in the treatment of recurrent and platinum‐resistant EOC. Miao et al reported that 29 patients with recurrent, platinum‐resistant, pretreated EOC were treated with 500 mg apatinib daily. The ORR was 41.4% and the DCR was 68.9% with a PFS of 5.1 months.^12^ In another study, the combination of apatinib and oral etoposide achieved 54% ORR, 86% DCR, and a median PFS of 8.1 months.^14^ Thereby, our data showed that apatinib treatment at 250 mg had similar efficacy in platinum‐resistant or platinum‐refractory EOC. DCR was 61.5% and the median PFS was 4.0 months, which is comparable to the previous study. Most of the patients were in the end‐stage of the disease in the present study, with limited therapeutic options due to primary or acquired chemotherapeutic resistance. These patients who had undergone multiple regimens and multiple courses of chemotherapy had poor physical condition and tolerance. Therefore, a lower dose of apatinib shows promise.

For platinum‐resistant/refractory ovarian cancer patients, on‐platinum single‐agent salvage chemotherapy is often used^18^ Overall response rates range from 10% to 35% in phase II studies, including paclitaxel 14%, docetaxel 20% to 35%, pegylated liposomal doxorubicin (PLD) 25.7%, topotecan 20.5%, gemcitabine 15% to 20%, and oral etoposide 25%.^19^ A randomized phase III trial compared the efficacy of gemcitabine to PLD in 195 platinum‐resistant ovarian cancer patients. Median PFS was 3.6 vs 3.1 months and median OS was 12.7 vs 13.5 months; ORR was 6.1% vs 8.3%.^20^ Comparing to these single‐agent chemotherapies, our results show superior ORR of low‐dose apatinib even in ITT analysis (27.7%) but similar PFS. Therefore, our results provided another non‐chemotherapy treatment choice for patients with platinum‐resistant/refractory ovarian cancer.

In consideration of the treatment‐related toxicity, we found that 250 mg was mostly chosen as the initial dose in practice. The toxicity of apatinib was largely tolerable and controllable in most previous studies. However, treatment‐related toxicity may reduce patients' treatment compliance and quality of life. A recent meta‐analysis showed the incidence of hypertension and proteinuria at all‐grade was 45.4% and 45.1%, and at high‐grade was 9.7% and 3.7%.^21^ The most common AEs of apatinib observed in the present study were also hypertension (19.2%) and hand‐foot syndrome (17.3%), which were lower than those reported in patients receiving a high dose (500 mg or more).^8^, ^12^, ^13^ In a phase II study, platinum‐resistant or platinum‐refractory ovarian cancer patients were administered with apatinib (500 mg/d) in combination with oral etoposide. Dose reductions occurred in as many as 82% of the patients, with 57% having one dose reduction and 43% needing two.^14^ In another phase II study of 500 mg apatinib for EOC, 39.3% of the patients had to interrupt the dose within 1 month, and 32.1% of the patients had to reduce the dose due to severe hand‐foot syndrome and hypertension.^14^ The high rate of dose reduction in the previous study may also indicate that 250 mg may be more acceptable. The relatively expensive cost of apatinib is the second reason why 250 mg was more widely used in practice.

So far, there is no reliable biomarker to measure the response of apatinib. Liu et al reported that the presence of hypertension, proteinuria, or hand‐foot syndrome in the first cycle of apatinib therapy in metastatic gastric cancer patients was a feasible biomarker for the evaluation of antitumor efficacy.^22^ However, our results did not show a clear association between AEs and efficacy. Lin et al showed that the older age group was independent predictors for prolonged PFS in metastatic breast cancer patients treated with apatinib.^23^ In the present study, the older age group also showed longer PFS and higher DCR. Angiogenesis is essential for tumor growth and metastasis, and a study showed that invasive breast carcinoma‐induced angiogenesis is related to age.^24^ The older age patients had lower vascularized areas,^24^ whereas low microvessel density was associated with longer OS and PFS in ovarian cancer patients, as reported by meta‐analysis.^25^ However, due to the small sample size and retrospective study lacking placebo control, the correlation between age and apatinib response may be a coincidence or maybe because of the longer PFS of untreated old patients. Regardless of the underlining mechanism, our results suggest that taking low‐dose apatinib is more acceptable especially in elderly patients without concession of efficacy.

The present study has several limitations. Firstly, as a retrospective study with a limited number of patients from one center, our study carries all the inherent limitations and biases associated with this. Secondly, because of the different populations enrolled, the efficacy rate is incomparable between our study and the previous one. A randomized study may be required to obtain a conclusion. Thirdly, 13 of 92 (17%) were excluded because they were lost to follow‐up or insufficient data were present. This may cause biases in the efficacy and safety analysis. To balance the selection bias, we conducted an ITT analysis of response rate and found the ORR of low‐dose apatinib in ITT analysis was lower than other studies of apatinib but still higher than single‐agent chemotherapy.

In conclusion, we recommend that an initial dose of 250 mg daily of apatinib is acceptable in the therapy of platinum‐resistant or platinum‐refractory EOC.

## COMPETING INTERESTS

The authors have no conflict of interest to declare.

## ETHICS APPROVAL AND CONSENT TO PARTICIPATE

The study was conducted in accordance with the Declaration of Helsinki and its amendments, and with Good Clinical Practice guidelines. Ethical approval for this study was obtained from the institutional review board at Fudan University Shanghai Cancer Center, Shanghai, China. The need for consent for this study was waived by the institutional review board (institutional review board at Fudan University Shanghai Cancer Center, Shanghai, China) due to its retrospective design.

## AUTHOR CONTRIBUTIONS

Conception and design: Wei Chen, Zhong Zheng, and Xiaohua Wu. Collection and assembly of data: Wei Chen, Ziting Li, and Zhong Zheng. Data analysis and interpretation: Wei Chen, Zhong Zheng, and Xiaohua Wu. Manuscript writing: Wei Chen and Zhong Zheng. Final approval of manuscript: All authors. Accountable for all aspects of the work: All authors.

## Data Availability

The data that support the findings of this study are available on request from the corresponding author. The data are not publicly available due to privacy or ethical restrictions.
